# Building on models—a perspective for computational neuroscience

**DOI:** 10.1093/cercor/bhaf295

**Published:** 2025-11-09

**Authors:** Hans Ekkehard Plesser, Andrew P Davison, Markus Diesmann, Tomoki Fukai, Tobias Gemmeke, Padraig Gleeson, James C Knight, Thomas Nowotny, Alexandre René, Oliver Rhodes, Antonio C Roque, Johanna Senk, Tilo Schwalger, Tim Stadtmann, Gianmarco Tiddia, Sacha J van Albada

**Affiliations:** Department of Data Science, Faculty of Science and Technology, Norwegian University of Life Sciences, PO Box 5003, 1432 Ås, Norway; Institute for Advanced Simulation (IAS-6), Jülich Research Centre, Wilhelm-Johnen Strasse, 52428 Jülich, Germany; Käte Hamburger Kolleg: Cultures of Research (co:/re), RWTH Aachen University, Templergraben 55, 52056 Aachen, Germany; Paris-Saclay Institute of Neuroscience, CNRS, Université Paris-Saclay, 151 route de la Rotonde, 91400 Saclay, France; Institute for Advanced Simulation (IAS-6), Jülich Research Centre, Wilhelm-Johnen Strasse, 52428 Jülich, Germany; JARA-Institute Brain Structure-Function Relationships (INM-10), Jülich Research Centre, Wilhelm-Johnen Strasse, 52428 Jülich, Germany; Department of Psychiatry, Psychotherapy and Psychosomatics, Medical Faculty, RWTH Aachen University, Pauwelsstrasse 30, 52074 Aachen, Germany; Department of Physics, Faculty 1, RWTH Aachen University, Otto-Blumenthal-Straße, 52074 Aachen, Germany; Neural Coding and Brain Computing Unit, Okinawa Institute of Science and Technology, 1919-1 Tancha, Onna-son, Kunigami-gun, Okinawa 904-0495, Japan; Chair of Integrated Digital Systems and Circuit Design (IDS), RWTH Aachen University, Templergraben 55, 52056 Aachen, Germany; Department of Neuroscience, Physiology and Pharmacology, University College London, Gower Street, London WC1E 6BT, United Kingdom; Sussex AI, School of Engineering and Informatics, University of Sussex, Chichester I Building, Falmer, Brighton BN1 9QJ, United Kingdom; Sussex AI, School of Engineering and Informatics, University of Sussex, Chichester I Building, Falmer, Brighton BN1 9QJ, United Kingdom; Chair of Computational Network Science, Faculty of Computer Science, RWTH Aachen University, Ahornstraße 55, 52074 Aachen, Germany; Department of Computer Science, University of Manchester, Kilburn Building, Oxford Road, Manchester M13 9PL, United Kingdom; Department of Physics, School of Philosophy, Sciences and Letters of Ribeirão Preto, University of São Paulo, Av. Bandeirantes 3900, Monte Alegre, Ribeirão Preto, SP, 14040-901, Brazil; Institute for Advanced Simulation (IAS-6), Jülich Research Centre, Wilhelm-Johnen Strasse, 52428 Jülich, Germany; Sussex AI, School of Engineering and Informatics, University of Sussex, Chichester I Building, Falmer, Brighton BN1 9QJ, United Kingdom; Institute of Mathematics, Technische Universität Berlin, Straße des 17. Juni 136, 10623 Berlin, Germany; Bernstein Center for Computational Neuroscience Berlin, Unter den Linden 6, 10099 Berlin, Germany; Chair of Integrated Digital Systems and Circuit Design (IDS), RWTH Aachen University, Templergraben 55, 52056 Aachen, Germany; Istituto Nazionale di Fisica Nucleare (INFN), Sezione di Cagliari, Department of Physics, Complesso Universitario di Monserrato, S.P. per Sestu – Km 0,700, 09042 Monserrato (CA), Italy; Institute for Advanced Simulation (IAS-6), Jülich Research Centre, Wilhelm-Johnen Strasse, 52428 Jülich, Germany; Institute of Zoology, University of Cologne, Albertus-Magnus-Platz, 50923 Cologne, Germany

**Keywords:** cortex, modeling, neuromorphic computing, sharing, simulation

## Abstract

Neural circuit models are essential for integrating observations of the nervous system into a consistent whole. Public sharing of well-documented codes for such models facilitates further development. Nevertheless, scientific practice in computational neuroscience suffers from replication problems and little re-use of circuit models. One exception is a data-driven model of early sensory cortex by Potjans and Diesmann that has advanced computational neuroscience as a building block for more complex models. As a widely accepted benchmark for correctness and performance, the model has driven the development of CPU-based, GPU-based, and neuromorphic simulators. On the 10th anniversary of the publication of this model, experts convened at the Käte Hamburger Kolleg Cultures of Research at RWTH Aachen University to reflect on the reasons for the model’s success, its effect on computational neuroscience and technology development, and the perspectives this offers for the future of computational neuroscience. This report summarizes the observations by the workshop participants.

## Introduction

The neural network model by Potjans and Diesmann (2014) represents the circuitry found under 1 mm^2^ of early sensory cortex; we refer to this model as PD14 for brevity. In the spirit of FAIR (findable, accessible, interoperable, reusable) and open science, the authors made PD14 available to the research community in multiple versions, including a version expressed in the simulator-agnostic language PyNN (Davison et al. 2009) that they shared on the Open Source Brain platform (Gleeson et al. 2019). Comprising some 77,000 neurons connected via about 300 million synapses, the model can be specified in fewer than 400 lines of Python code (excluding documentation) and can be simulated on modern laptops, although systematic exploration benefits from use of compute clusters.

On the occasion of the 10th anniversary of the publication, experts from three continents, ranging from early post-doc to senior scientist and from computational neuroscientist to electrical engineer, convened at the Käte Hamburger Kolleg Cultures of Research at RWTH Aachen University from April 3 to 4, 2024, to assess the impact of PD14 on the practice and culture of research in computational neuroscience and related disciplines.

**Fig. 1:**
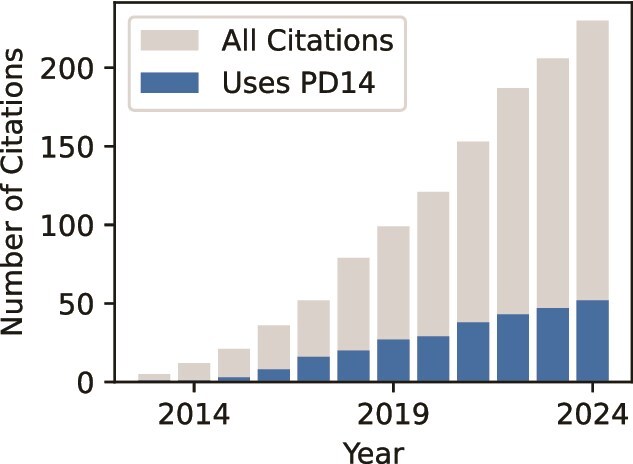
Citations of the PD14 model. Gray (lighter) bars: cumulative number of citations in peer-reviewed publications, including references to the online-first version published in *Cerebral Cortex* in December 2012. Blue (darker) bars: cumulative number of articles using the model. The model code and bibliographic data curated by the authors are available (https://github.com/INM-6/microcircuit-PD14-model).

Hans Ekkehard Plesser opened the workshop with a brief review of spiking simulation history since the pioneering work by Farley and Clark (1954). Sadly, in spite of an urgent call by De Schutter (2008) for more systematic model sharing using common tools, Senk et al. (2022) found that roughly half of neuronal network models surveyed on ModelDB (McDougal et al. 2017) and Open Source Brain (Gleeson et al. 2019) were still implemented in general-purpose programming languages such as MATLAB, Python, or C, even though modeling in computational neuroscience allows for a clear separation between the specific scientific model and the generic brain simulator (Einevoll et al. 2019). It is this separation that enables the representation of PD14 by a short script using high-level concepts from the neuroscience domain. Nevertheless, current research practice in computational neuroscience still appears to be characterized by a lack of model sharing in the sense of researchers actively building new models based on model implementations by others. The PD14 model is a rare exception to this pattern (Fig. 1): As of March 2024, 52 peer-reviewed studies using the model as building blocks had been published and 233 had cited it.

**Fig. 2:**
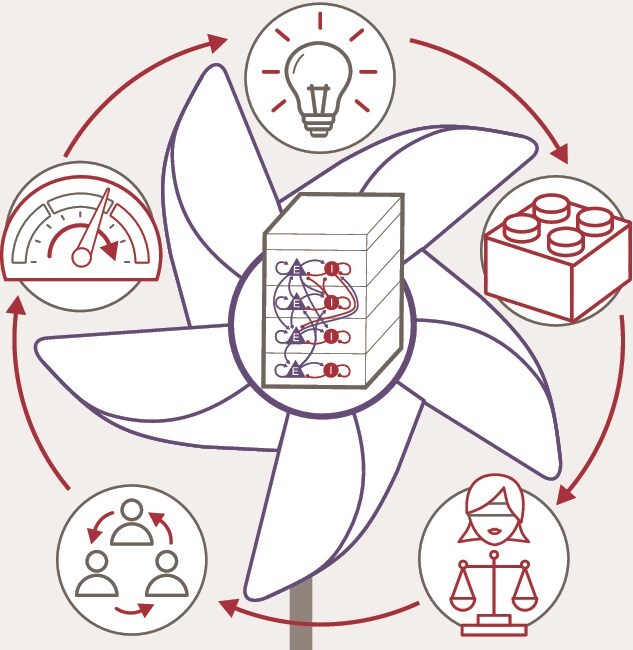
Impact of the PD14 model. The model of the cortical microcircuit (center) had an impact on five aspects of research (clockwise from top): conceived to provide neuroscience insight, the model became a building block of more advanced models, served as reference for the validation of mean-field theories, drove the development of methods for model sharing, and became a standard for benchmarking of neuromorphic and GPU systems.

While the PD14 model was originally conceived in 2006 to gain deeper neuroscientific insights into the relation between network structure and dynamics, it soon stimulated research in multiple ways as illustrated in Fig. 2. First of all, scientists started to use PD14 as a building block for more complex brain models; some examples are discussed in section PD14 as a Building Block. Second, the model became popular as a testbed for the validation of mean-field analyses of network dynamics as detailed in section PD14 as a Reference. Third, PD14 played an important role in shaping approaches to model sharing as described in section PD14 Driving Model Sharing, as participants in the large-scale European neuroscience project FACETS (2005 to 2010, https://facets.kip.uni-heidelberg.de) realized that a common language was required to reliably transfer network models from one simulation engine to another, including neuromorphic engines. Early versions of PD14 represented a challenging test case for the emerging PyNN language, and the model later served as a test case for the documentation and curation of complex model code on platforms such as Open Source Brain to facilitate re-use. These efforts on model sharing were furthered by the subsequent large-scale projects BrainScaleS (2011 to 2015, https://brainscales.kip.uni-heidelberg.de) and Human Brain Project (2013 to 2023, https://www.humanbrainproject.eu) and through participation in the project preparing for the use of the K computer in Japan (Diesmann 2013). Finally, the model has helped to push the boundaries of simulation technology as a key benchmark for neuromorphic systems as summarized in section PD14 as a Neuromorphic Benchmark and recently reviewed in detail by Senk et al. (2025).

Plesser challenged the participants to discuss why the PD14 model has been re-used by other researchers and on this example to elucidate what it takes for a model to be re-used not only in computational neuroscience but across disciplines.

## A digital twin for the cortical microcircuit

Markus Diesmann reported that work on the model started in May 2006 at the Bernstein Center for Computational Neuroscience at the Albert-Ludwigs-University in Freiburg, Germany, and came to fruition between October 2006 and the spring of 2011 at the RIKEN Brain Science Institute in Wako-shi, Japan. The model was first presented publicly at the Annual Meeting of the Society for Neuroscience in 2008 (Potjans and Diesmann 2008) and was first used as a benchmark case by external researchers in 2010 (Djurfeldt et al. 2010). The scientific paper describing the model and its anchoring in anatomical and physiological data was completed at Jülich Research Centre in the spring of 2012 and published online ahead of print as an open-access publication in December 2012. Due to production delays common at the time, the paper did not appear in print in *Cerebral Cortex* before March 2014.

An executable version of the PD14 model was released in June 2014 as part of NEST 2.4.0. This version was formulated in the simulation language SLI (Diesmann et al. 1995). Just a month later, an implementation of PD14 in the simulator-independent network specification language PyNN was published as part of the PyNN code base (https://github.com/NeuralEnsemble/PyNN/commit/4f09b4ef05e117af6404e5ff5f7b6568dd5ba2b0), allowing for model exploration via Python, which was rapidly becoming the programming platform of choice in computational neuroscience (Muller et al. 2015). A Python implementation using NEST’s native PyNEST interface (Eppler et al. 2009) followed with NEST 2.12.0 in March 2017 and since December 2017 the model has been available on the Open Source Brain platform (https://v2.opensourcebrain.org/repositories/368).

Diesmann pointed out that work on the model started in the mid-2000s, a decade after the seminal work by van Vreeswijk and Sompolinsky (1996) and Amit and Brunel (1997) established the value of networks operating in a regime of balanced excitation and inhibition. At this time, work on the dynamics of such networks focused on two-population models guided by a fundamental construction principle of the local network in a cortical layer (Brunel 2000). The local cortical network is attractive for experimentalists and theoreticians to this day because it exhibits a dual universality: (i) In the development of mammals, the volume of the brain increased by three orders of magnitude from mouse to human, but the structure of the local cortical network below a square millimeter patch of surface remained largely unchanged; (ii) the structure is to a large extent independent of whether a cortical area processes auditory, visual, or tactile information, or is involved in motor planning (see the section Building Block for Models with Larger Explanatory Scope for the nature of differences). This universality gives natural scientists confidence that there are fundamental principles to be discovered. PD14 was not the first nor the last model attempting to capture key properties of the local cortical circuit. An influential review by Douglas and Martin (2004) made the notion of the canonical microcircuit known to a wide audience. Further studies reproduced complex dynamical features like the transition between sleep and wakefulness (Bazhenov et al. 2002; Hill and Tononi 2005) and the occurrence of oscillatory phenomena (Traub et al. 2005) or concentrated on information processing capabilities (Haeusler and Maass 2007; Bastos et al. 2012; Habenschuss et al. 2013).

Digital twins as digital representations of the elements and interactions making up a piece of nature or technology have been widely adopted in neuroscience over the past decade (Amunts et al. 2024). In line with this development, several studies have been published since 2010, aiming at the integration of anatomical and physiological data and the simultaneous reproduction of a range of network-level observations. A number of these models attempt to represent all the neurons and synapses in the volume of cortex they describe to enable multi-scale investigations and avoid the risks of distortions of activity by down-scaling (van Albada et al. 2015). Markram et al. (2015) presented a model based on multi-compartment model neurons, while Antolik et al. (2024) restricted the single-neuron dynamics to point-neuron models but covered a cortical area of 5 × 5 mm^2^ of cat primary visual cortex with a neuron density scaled down to 10%. Finally, Billeh et al. (2020) studied the mouse primary visual cortex using cell-type-specific dynamics. All these studies made their models available as open source and are based on well-established open-source simulation codes. In the light of these developments in the community, Diesmann entitled his historical account “A digital twin for the cortical microcircuit.”

Diesmann elaborated that the primary research question motivating the construction of the PD14 model was to understand how the actual structure of the cortical network shapes the observed network dynamics. To achieve this, the model starts from strong hypotheses about the architecture of cortex such as the organization of the nerve cells into four layers, each containing an excitatory and an inhibitory neuron population (model sketch in Fig. 2). To reduce the influence of the single-neuron dynamics to a minimum, single neurons are represented by point-neuron models identical across all populations. The only property distinguishing inhibitory neurons from excitatory neurons is that their outgoing synapses are inhibitory with a weight increased by a constant factor. Apart from this factor, all synaptic weights are drawn from the same distribution (with specific exceptions). What was left to specify were the numbers of neurons in each population and the 8 × 8 connection probabilities. The size of the model was chosen such that the majority of the local synapses of a neuron are captured and parallel to the cortical surface the network can still be considered as randomly connected. This resulted in a surface area of a square millimeter. Diesmann concluded his historical review by remarking that PD14 was essentially constructed by a single doctoral student and may mark a crossing point in the sense that constructing larger and more complex models from scratch will require the collaboration of larger groups of researchers.

## PD14 as a building block

### Re-use for a model of attention

Cortical microcircuits are a fundamental processing unit with a stereotyped multilayer circuit structure consisting of excitatory cells and several subtypes of inhibitory cells. These neural circuits receive bottom–up input from the peripheral sensory regions in layer 4 and top–down inputs from higher cortical areas in superficial and deep layers. They are thought to be pivotal in processing bottom–up sensory and top–down attention signals. The PD14 model allows us to test how these two qualitatively different types of inputs interact within cortical microcircuits to perform visual processing. In the visual cortex, the spatial and feature-based modes of attention are known to influence visual processing differently (McAdams and Maunsell 1999; Reynolds et al. 1999; Martinez-Trujillo and Treue 2004; Ling et al. 2009). Tomoki Fukai reported that as part of RIKEN’s K computer project (https://www.riken.jp/en/collab/resources/kcomputer/), Wagatsuma et al. constructed a minimal model of the visual cortical circuit by laterally connecting two PD14 models to explore the differential effects of spatial- and feature-based attention on the processing of orientation selectivity of visual cortical neurons (Wagatsuma et al. 2011; Wagatsuma et al. 2013). Their model simultaneously accounted for the multiplicative scaling of neuronal responses in spatial attention and additive modulations of orientation tuning curves in feature-based attention. Furthermore, the model predicted contrasting differences in attentional modulations of neural responses between different cortical layers.

Next, Fukai discussed the generation mechanisms of gamma (30 ~ 80 Hz) and beta (20 ~ 30 Hz) oscillations in a local cortical circuit model. The Potjans–Diesmann model has the virtue of representing all cortical layers and biologically realistic connectivity structures but only possesses pyramidal cells and one subtype of inhibitory neurons, parvalbumin (PV) interneurons. Therefore, the model is still oversimplified compared to cortical microcircuits. A refined microcircuit model involves PV, somatostatin (SOM), and vasoactive intestinal polypeptide (VIP) interneuron subtypes, which allows for investigations of attentional modulations of different interneuron subtypes (Wagatsuma et al. 2023). However, this model does not distinguish the cortical layers, and differential attentional modulations across cortical layers therefore cannot be explored. Their results suggest that inhibitory signals from PV and SOM neurons preferentially induce neuronal firing at gamma (30 ~ 100 Hz) and beta (20 ~ 30 Hz) frequencies, respectively, in agreement with observed physiological results. Furthermore, the model predicts that rapid VIP-to-SOM inhibition underlies marked attentional modulation at low-gamma frequencies (30 ~ 50 Hz) observed by Womelsdorf et al. (2006). Altogether, results suggest distinct and cooperative roles of inhibitory interneuron classes in visual perception and show the potential of large-scale simulation models of the brain’s neural networks for exploring neural dynamics related to cognitive functions.

### Re-implementing and modifying PD14

Antonio Roque started his presentation by referencing his group’s earlier efforts to replicate spiking activity patterns in large-scale cortical models. These initial studies were hampered by rudimentary models that failed to accurately represent the intricate details of cortical connectivity. The advent of the PD14 model marked a significant advancement in their research. This model alleviated the burden of reconstructing the anatomy of a cortical column, a task previously insurmountable for smaller groups, by providing an accessible and reliable graph structure for the development of models for the study of cortical dynamics. Roque highlighted that a key feature of the PD14 model is its thorough and standardized documentation, facilitating its ready application.

Roque detailed the various adaptations of the PD14 model developed in his laboratory. These adaptations maintain the same random graph structure of the original model, connecting eight populations of excitatory and inhibitory neurons. However, in contrast to the original model, where both neuron types were represented by the same leaky integrate-and-fire neuron model, the variants employ different neuron models. Examples include adaptive exponential integrate-and-fire neurons to represent neurons from different electrophysiological classes (Kamiji et al. 2017; Shimoura 2021) and stochastic neurons (Roque et al. 2017; Cordeiro 2019). The addition of population-specific neuron types had a significant impact on the spontaneous activity of the network. However, the introduction of stochasticity did not produce notable deviations from the deterministic model.

Roque also discussed two distinct replications of the PD14 model: one implemented in Brian 2 (Shimoura et al. 2018) and another in NetPyNE (Romaro et al. 2021), the latter accompanied by a recipe for scaling the model’s size. He emphasized that replicating complex models such as PD14 offers an invaluable training experience for students, exposing them to the systematic and rigorous methodology behind these models. Roque advocated for the inclusion of such replication tasks in the fundamental training of students enrolled in computational neuroscience courses.

### Building block for models with larger explanatory scope

The PD14 model forms a natural building block for multi-area models of cortex when the detailed properties of the microcircuits are adapted to the peculiarities of each area. Sacha van Albada discussed how such multi-area models can be used to link local and global cortical connectivity to multi-scale resting-state dynamics. This work investigates how certain aspects of cortical dynamics, not captured by local models such as PD14, can emerge from interarea interactions: a spectrum of spiking activity with enhanced power at low frequencies (Chu et al. 2014), propagation of activity predominantly down the visual hierarchy (Dentico et al. 2014), and a pattern of interarea correlations as seen in resting-state BOLD fMRI (blood-oxygen-level-dependent functional magnetic resonance imaging). These dynamical features were addressed in a model of all vision-related areas in one hemisphere of macaque cortex (Schmidt et al. 2018a, 2018b); code is provided on GitHub (https://github.com/INM-6/multi-area-model). In this model, the relative indegrees of the different population pairs were kept the same as in PD14 while adjusting the population sizes to the macaque areas and layers. This choice was motivated by the strong role of indegrees in determining firing rates and the aim to keep the relative firing rates of the layers and populations similar to those in PD14. However, achieving reasonable firing rates required small adjustments in the model’s connectivity, informed by mean-field theory (Schuecker et al. 2017). Increasing the strength of the cortico-cortical synapses, particularly onto inhibitory cells, then brought the model close to a transition between a low-activity and a high-activity state, with population bursts propagating across areas.

While the multi-area model goes a long way toward reproducing the aforementioned aspects of cortical dynamics, it still has shortcomings that were addressed in a refined version of the model (Pronold et al. 2024a). Following a motor cortex model of Rostami et al. (2024), this refined model divides each layer and area into joint clusters of excitatory and inhibitory neurons. The enhanced local balance afforded by this change supports plausible firing rate distributions in all areas, where the Schmidt et al. (2018b) model still had populations with vanishing or excessive firing rates. In addition, the E/I clustering enables both feedforward and feedback activity propagation that is much more robust and removes the strong synchrony inherent in the population bursts of the Schmidt et al. (2018b) model. The Pronold et al. (2024a) model moreover reproduces the experimentally observed quenched neural variability upon visual stimulation (Churchland et al. 2010).

A further multi-area model presented by Sacha van Albada encompasses all areas in a coarse parcellation of a human cortical hemisphere (Pronold et al. 2024b). Just like the macaque models, it represents each area by a 1 mm^2^ microcircuit with the full density of neurons and synapses. However, in this work, the authors chose to maintain the relative local connection probabilities as opposed to the indegrees of the PD14 model. The reasoning was that certain areas have thin layers (particularly layer 4) with very few neurons that would otherwise nevertheless have to supply large numbers of synapses. Maintaining the relative connection probabilities, this problem is eliminated, as the numbers of synapses are in this case approximately proportional to the numbers of neurons. As opposed to the macaque model, the human cortex model did not require a mean-field-based stabilization to achieve a reasonable low-rate state. In addition, the experimentally observed spiking statistics and pattern of interarea correlations are reproduced in a stable low-rate state instead of just below a transition to a high-rate state. Matching the macaque model, experimentally observed activity is well captured when the cortico-cortical synapses are stronger than the local ones. The code for the Pronold et al. (2024b) model is also freely available (https://github.com/INM-6/human-multi-area-model).

## PD14 as a reference

### A detailed anatomical model as driver of analytical coarse-grained descriptions

The PD14 model is a microscopic model in the sense that each neuron of a large neural network is modeled (and simulated) individually. While microscopic models can be related to and constrained by measured biophysical parameters, the inherent complexity of these high-dimensional systems precludes a full theoretical understanding of the emerging dynamical behavior. Moreover, in the case of performance-critical simulations such as the exploration and fitting of parameters or large-scale brain simulations, a detailed microscopic simulation of the network resolved at the level of individual neurons may not be fast enough—and not be necessary. Instead, coarse-grained, mesoscopic or macroscopic mean-field models are efficient to simulate and amenable to mathematical analysis. To maintain the link to biophysical parameters and to be consistent across scales, meso- and macroscopic models need to be derived “bottom–up” from—and tested against—a realistic microscopic model.

Tilo Schwalger noted that the PD14 model has ideal properties for this purpose. On the one hand, as a reference implementation of a cortical column, PD14 provides the biologically relevant microscopic foundation for theoretical analysis. On the other hand, the model features convenient mathematical properties that make it ideally suited for a mean-field reduction: It is structured as a network of roughly homogeneous neuronal populations, and it is based on integrate-and-fire model neurons. However, the PD14 model also exposes challenges for a mean-field theory of cortical circuits, notably the fluctuations caused by the finite number of neurons and the fast nonstationary dynamics governed by single-neuron dynamics with refractoriness. Fast nonstationary dynamics of population activities can be obtained from the dynamics of the membrane-potential distributions of interacting neuron populations. A numerically efficient method to evolve distributions of membrane potentials forward in time is the DiPDE method (partial differential equations with displacement, Iyer et al. 2013). Cain et al. (2016), Schwalger et al. (2017), and Osborne et al. (2022) returned to PD14 to demonstrate the capabilities of generic simulation code for multi-dimensional population density models such as MIIND (de Kamps et al. 2008) and show correspondence of the results with DiPDE in the transient and stationary spike rates.

Schwalger presented a mesoscopic theory (Schwalger et al. 2017) that starts from a network of generalized integrate-and-fire (GIF) neurons—a neuron model that reliably reproduces the spiking responses of real cortical cells and whose parameters are catalogued in the Allen Cell Types Database for various cell types (Teeter et al. 2018). The mesoscopic mean-field dynamics is given by a stochastic integral equation that precisely captures finite-size fluctuations and fast nonstationary dynamics. Applied to a variant of the PD14 model based on GIF neurons, the numerical implementation of the mesoscopic model yields a speed-up of about 150 times compared to the corresponding microscopic simulation. The central object in the theory is a hazard rate function, which is not present in the original PD14 model driven by external Poisson noise. However, several approximations are available (Schwalger 2021), enabling an efficient mesoscopic mean-field description also for the original PD14 model. Finally, Schwalger explained how the mesoscopic dynamics can be further reduced to highly efficient, low-dimensional stochastic dynamics based on an eigenfunction expansion.

### Model epistemics: how to select models in the face of data

Large models like PD14 have a large parameter space and thus also a large functional space. If ultimately the goal of these models is to study neural circuits, it is important to select parameters that actually represent those circuits—to ensure that studies are scientifically relevant and that simulators are benchmarked in scientifically relevant regimes.

One challenge in this context is that many different combinations of parameters may nevertheless define models that are functionally very similar—a situation described as “epistemic uncertainty,” ie uncertainty in the model (Kiureghian and Ditlevsen 2009; Hüllermeier and Waegeman 2021). With modern machine learning tools, it is increasingly possible to find large ensembles of candidate models, each the local optimum of some loss. Alexandre René showed that this indeed occurs when we fit a mesoscopic model according to Schwalger et al. (2017) to a reduced variant of the microscopic PD14 model (René et al. 2020). However, not all of the local optima found correspond to equally good candidate models; we would like to focus research efforts on those models that are best supported by the data.

Key to this goal is precisely defining what we mean by “models” and “best supported.” Typically, in neuroscience (and in science more broadly), models consist of both a set of equations and a set of parameters; the best models are those that not only reproduce the data observed during an experiment but can also predict observations expected in replications or new experiments. As René and Longtin (2025) explain, this is at odds with the assumptions underlying most statistical methods of model selection. First, classic methods such as the Akaike and Bayesian Information Criteria (Akaike 1973; Schwarz 1978) assume that the model will be refit to every new dataset: They do not compare specific sets of parameters but rather entire families of models. In other words, they compare the global optima of models defined by different equations and are inappropriate for comparing local minima of models defined with the same equations. Second, even a method like the Widely Applicable Information Criterion, which compares the expected loss over replications (Vehtari et al. 2017), still assumes that the data-generating process is perfectly reproducible—an unrealistic assumption for real-world experiments.

To identify the best parameter sets for a neural circuit, we need a comparison method that accounts for the variability of experiments across replications. René presented a method (René and Longtin 2025) that does so by applying the following principle: Candidate models represent the part of the experiment that we understand and can replicate. Concretely, the discrepancy between model predictions and observations is used to predict the uncertainty on a model’s expected loss, also known as the risk. The result is that to a model *A* is associated a distribution of risk values ${R}_A$, representing the expected loss across replications. A model *B* can be confidently rejected by *A* if it has a higher risk across almost all replications.

## PD14 driving model sharing

### Platforms and standards for sharing

Padraig Gleeson presented initiatives related to model sharing and standardization in neuroscience, as well as the ways these have been used to make PD14 more accessible to modelers and to manage and maintain the multiple versions of the model that have been produced. The Open Source Brain platform (OSB, http://www.opensourcebrain.org) is an initiative to encourage the open sharing and collaborative development of models in computational neuroscience (Gleeson et al. 2019). The source files for OSB models are hosted on code-sharing platforms like GitHub (which provides all the standard functionality required for open-source development like change history tracking, issue management, and user authentication), and the main OSB website lists and links to a number of these model repositories, allowing users to search for models of interest. OSB also provides additional neuroscience-specific tools for visualizing, simulating, and analyzing them.

The OSB repository for PD14 available on GitHub (https://github.com/OpenSourceBrain/PotjansDiesmann2014) provides versions of the model developed in the original NEST SLI scripts, in PyNEST Python scripts, and an implementation of the network in the PyNN modeling language (Davison et al. 2009; see section Disentangling Model from simulation), as well as an export of the model to NeuroML 2 (Cannon et al. 2014), a simulator-independent model description language based on the extensible markup language (XML). The original version of the OSB platform (version 1) contains a project that is linked to this GitHub repository (https://v1.opensourcebrain.org/projects/potjansdiesmann2014) and allows the user to load and visualize the NeuroML version of the model and analyze the connectivity (Fig. 4 of Gleeson et al. (2019)), as well as to run simple simulations. The more recently developed OSB version 2 of PD14 (https://v2.opensourcebrain.org/repositories/368) links to the same GitHub repository and so will always refer to the latest version of the code. It offers additional functionality such as executing the model in a browser-based, interactive computing environment based on JupyterLab.

Gleeson explained that, in addition to the primary goal of making the PD14 model more accessible for the community using OSB, working with such a robust and well-maintained model has helped test the underlying infrastructure for model development, testing, and management on the platform. An example of this is the OSB Model Validation framework (OMV, Gleeson et al. (2019)) for testing and validating consistent behavior of model code across multiple versions of simulators, Python releases, or other dependencies. The OMV tests a model upon each change to the code in the GitHub repository. This helps check that the model runs identically across new releases of NEST, for example. These developments demonstrate how a computational model of widespread usage and interest within the community can have benefits beyond the primary scientific ones. It provides a concrete use case for testing infrastructure and platforms for model sharing and collaborative development, and the tools and processes developed for this purpose can be reused for many other computational models of neuronal systems.

### Disentangling model from simulation

Andrew Davison presented a brief history of model-sharing efforts in neuroscience, starting with BABEL (http://www.genesis-sim.org/BABEL/), the user group for the GENESIS simulator (Bower and Beeman 1998), and covering ModelDB (https://modeldb.science/), NeuroML (Gleeson et al. 2010), Neosim (Howell et al. 2003), OSB, EBRAINS (https://search.kg.ebrains.eu) and GitHub (https://github.com/). This long history is illustrative of the many technical and sociological challenges in sharing and building on models. Davison highlighted the progress that has been made, including the development and increasing uptake of declarative and other simulator-independent model specifications, better tools for running simulations and for managing code versions, and the spread of a culture of open science and open-source software, and enumerated some of the remaining challenges. As an example of how PD14 contributed to the progress already made in model sharing, it provided a challenging use case for the PyNN modeling language (Davison et al. 2009). Arising from the need in the European FACETS project for a simulator-independent model description language, PyNN facilitates model sharing by making it much easier to transfer models from one simulation engine to another, and to cross-verify different implementations. In developing the PyNN API and its implementation for different simulation engines, PD14 was a challenge that helped shape the way that complex networks are represented, such that the backend simulation engine or neuromorphic computing system has sufficient freedom for efficient optimization and parallelization.

Among the remaining challenges, Davison focused on a general lack of clear interfaces in most shared code for models of neural circuits. Most shared “models” are actually a mixture of model building code, simulation experiment code, data analysis code, and plotting code. This makes it more difficult to build upon or reuse such “models,” as only the model building code, and perhaps some of the simulation experiment code, is actually needed for reuse, and the details of how to connect the model as a component to other models, how to specify new stimuli/experimental protocols, or how to handle and interpret output data, are different for each model. As an example of this, for the NEURON simulator, files written in the NEURON Modeling Language (NMODL) are much more widely reused than Hoc or Python files, as they explicitly encapsulate a model component, with a well-defined interface.

The importance of specifying clear and clean interfaces as a way to disentangle different components of a software system is commonplace in programming and software engineering. In neuroscience and in systems biology, the need for clear boundaries as implemented through declarative model description formats was recognized with the creation of NeuroML, SBML (Systems Biology Modeling Language, Hucka et al. 2003), CellML (Cuellar et al. 2003), and SED-ML (Simulation Experiment Description Markup Language, Waltemath et al. 2011) in the early 2000s. Yet the majority of shared models in neuroscience still lacks clean interfaces.

In presenting possible pathways toward improving this situation, Davison highlighted the education of young researchers: When teaching about modeling and simulation, we should emphasize reusability and the FAIR principles (Wilkinson et al. 2016) and make students aware of the available tools.

Davison then presented a proposal for defining standardized interfaces for models. This proposal builds on the SciUnit framework (Omar et al. 2014) and its concept of capabilities and proposes to: (i) generalize the interface definitions away from use of the Python language, using a language-independent interface definition; (ii) develop a repository or library of standard capabilities, covering different levels of model abstraction and scope.

Models written in a given language such as Python would expose their functionality by implementing the capability interfaces. For declarative model definitions such as NeuroML (https://github.com/NeuroML/pyNeuroML), the capability interfaces would only need to be implemented once per language (eg in PyNeuroML) rather than once per model. Code to define simulation experiments and analysis pipelines would only interact with a model through these interfaces, which would then make it much easier to define and implement new experiments. The library of standard capabilities would also serve as a controlled metadata vocabulary for describing models in databases.

To illustrate this proposal, Davison finished by presenting a worked example using the PD14 model. The current version of the code combines model specification and simulation experiment specification, each with a separate parameter file. Several variants of the simulation experiment are available: For example, the network may either receive random spikes to represent parts of the brain outside the scope of the model or just corresponding direct currents. To implement these variants, a number of interfaces are needed, defined by capabilities such as “Receives-Poisson-Spikes” or “Produces-Spikes” for each cell type or cortical layer in the model. This suggests that composing more complex capabilities from basic building blocks may be needed.

In conclusion, Davison proposed that, while continuing to promote the use of declarative model formats, the community of neuroscientists interested in sharing and building on models should collaborate to develop a library of standardized model interfaces, inspired by SciUnit, that decouple programmatic model definitions from the surrounding code for simulation, stimulation, data management, analysis, and visualization. Such interfaces could be implemented as lightweight wrappers around existing programmatic model definitions. This would increase the reusability of models and be part of a roadmap toward a component-based, constructive computational neuroscience. Such standard interfaces could also be used as metadata in model-sharing databases, increasing the FAIRness of computational models more generally. To obtain a broad uptake, this effort would need collaborators from across a wide range of model scopes from subcellular to whole-brain and model abstraction levels such as detailed biophysical models, simplified spiking models, or mean-field models.

## PD14 as a neuromorphic benchmark

The relevance of PD14 as a benchmark for neuromorphic computing systems became apparent in the course of the European BrainScaleS project (2011 to 2015). After an exploratory phase, a range of demonstrators was selected and ultimately PD14 became Demo 1.1 of BrainScaleS (Meier 2015). Besides the neuroscientific concept of the microcircuit as a fundamental building block of cortex (see section A Digital Twin for the Cortical Microcircuit), the model also constitutes an elementary unit from the perspective of simulation. PD14 represents the minimal network size at which each neuron is supplied with the majority of the natural number of local synapses, and, at the same time, connection probability remains at the value observed in nature. From the size of PD14 upward, the total number of synapses in a network, and thus memory consumption, grows only linearly with network size while connection probability declines (Lansner and Diesmann 2012). For smaller networks, in contrast, synapse numbers and memory footprint typically scale quadratically. Thus, larger networks should be comparatively easy to simulate. A similar argument holds for the frequency of communication in a distributed simulation. Within the microcircuit, synaptic delays are in the submillisecond regime. In larger networks, the interaction of neurons from more distant parts of the network has larger delays, leaving a simulation engine room for optimization. PD14 thus defines an important challenge for neuromorphic computing, as once local networks of this size can be simulated, larger models should follow with relative ease.

The first direct comparison of simulation results for PD14 between a software simulation code and a neuromorphic system (van Albada et al. 2018) introduced “time to solution” and “energy per synaptic event” as measures of performance. The availability of these two simple quantitative measures contributed to the success of the model as a benchmark. In addition, the study introduced a set of metrics for the verification of the correctness of the simulation and a corresponding compact graphical representation. Subsequent publications used the same metrics and display the data in the same way. This approach offers researchers an established template for performance evaluation and reduces the uncertainty whether a reviewer would find the measures and representation adequate. As a side effect, results are easily comparable across studies.

### Optimizing for accuracy and efficiency

Johanna Senk started her presentation with a seemingly naive question that she and colleagues had asked a couple of years ago: If a simulation of the same neural network model is run both on a high-performance computing system (HPC) using NEST and on the neuromorphic hardware system SpiNNaker, are the results the same (Senk et al. 2017)? They took on the challenge, and they gradually became aware of its breadth and depth (van Albada et al. 2018). Due to inherent differences between simulators regarding algorithms, number representations, or random number generators, the simulated activity data for PD14 can only be compared on a statistical level. To verify correctness, the authors therefore compared distributions of firing rates, coefficients of variation of interspike intervals, and Pearson correlation coefficients. Although the resulting study (van Albada et al. 2018) eventually achieved a good match between the simulation results, at that time, neither technology enabled real-time simulation, and the required power exceeded the demands of the natural brain by several orders of magnitude.

The study was soon picked up by others, and subsequent simulations of the same model using and advancing different technologies, including graphics processing units (GPUs) and field-programmable gate arrays (FPGAs), have brought a performance gain for the community (Knight and Nowotny 2018; Golosio et al. 2021; Knight et al. 2021; Heittmann et al. 2022; Golosio et al. 2023; Kauth et al. 2023a), as detailed in sections GPU-accelerated Simulations and The Making of neuroAIx. Within just a few years, creative algorithmic strategies have been developed for making the best use of the respective systems, and the milestone of simulation in real time has been reached and surpassed at a significantly reduced energy consumption by all systems discussed in the workshop (Rhodes et al. 2019; Kurth et al. 2022). Senk pointed out that during this time, researchers also advanced the understanding of the mechanisms governing the model dynamics (see section A Detailed Anatomical Model as Driver of Analytical  Coarse-grained Descriptions) which is also informative for porting the model to different simulation platforms. For example, an analysis of the measures of network activity used for verification revealed the crucial role of the observation duration and heterogeneity in the neuron input (Dasbach et al. 2021). Another example is a recently developed toolbox for mean-field theory that provides access to methods for exploring the origin of network oscillations (Bos et al. 2016) and other analytical tools applicable to models such as PD14 (Layer et al. 2022).

Senk and colleagues considered the increasingly systematic benchmarking endeavors around the PD14 model (Albers et al. 2022) as a starting point for the co-development of simulation technologies and increasingly sophisticated neuroscientific models. Continuing these efforts, the performance of the more complex and demanding multi-area model is currently being assessed following the example of the PD14 model (see section Building Block for Models with Larger Explanatory Scope) (Schmidt et al. 2018b; Knight and Nowotny 2021; Tiddia et al. 2022).

### Modeling on neuromorphic hardware

Neuromorphic systems seek to take inspiration from the brain to develop novel computational devices and algorithmic paradigms for next-generation computers. Oliver Rhodes presented one such neuromorphic platform, the spiking neural network architecture *SpiNNaker* (Furber et al. 2013), developed at the University of Manchester, UK. The system can be scaled to connect 1 million programmable ARM cores via a brain-inspired multi-cast routing fabric, enabling real-time simulation of large-scale spiking neural network models. However, evaluating the performance of a neuromorphic architecture such as SpiNNaker is a distinct challenge, as the custom hardware system lacks the well-defined computing stack of modern CPU/GPUs. This means optimizing performance for specific applications is less well understood and ultimately requires full-stack development, from how the problem is defined to how it is mapped to the underlying computing and communication resources. Benchmark models are therefore important research tools to help evaluate and compare the performance of neuromorphic systems, and the neuroscience heritage and scale of the PD14 model made it an interesting benchmark to explore the performance of the SpiNNaker system.

The work presented by van Albada et al. (2018) demonstrated the first successful simulation of the PD14 model on SpiNNaker, representing the first time the model had been executed on neuromorphic hardware. Numerical accuracy was validated on the fixed-point arithmetic system; however, it was found that this required a reduced simulation timestep relative to the original system design (0.1 ms rather than 1 ms), which led to a slowing down of simulations. Furthermore, avoiding spike losses during transient initial synchronization of the model necessitated further slowing down so that the cortical microcircuit model was simulated at 20× slow-down relative to real-time (0.1 ms of simulation time simulated in 2 ms wall-clock time). In terms of energy, SpiNNaker computed the solution using 5.9 μJ per synaptic event. This figure closely matched that for an optimized HPC-simulated version of the model (5.8 μJ per synaptic event); however, simulation speed was almost an order of magnitude lower (the NEST + HPC version of the model executed at 3× slow-down relative to real time).

Using the lessons learned in the study above (van Albada et al. 2018), a subsequent research project was initiated to explore optimal mapping of the cortical microcircuit model to the SpiNNaker platform (Rhodes et al. 2019). This initiative was further boosted by a newly developed data loading approach, where individual processing cores could now be used to generate and initialize their own simulation data locally (and in parallel), rather than generating all data on a remote host and loading the data to the machine. This made better use of the massively parallel hardware and cut simulation initialization times from around 8 hours (van Albada et al. 2018), to around 8 minutes. This, in turn, facilitated wider exploration of the simulated model and better understanding of how it was interacting with the underlying processing and routing hardware. It was found that the main driver of simulation speed was the total number of spikes received by processing cores in a particular timestep.

Based on these insights, an alternative processing architecture was developed (Rhodes et al. 2019), deploying an ensemble of SpiNNaker cores to work in parallel, with cores specialized for specific tasks: updating neurons, processing incoming spikes, and generating stochastic inputs. This heterogeneous ensemble configuration achieved the first real-time simulation of the cortical microcircuit model, surpassing state-of-the-art results (at the time) simulated on HPC systems and GPUs, and achieving energy figures of 0.6 μJ per synaptic event, an order of magnitude reduction in energy use.

The PD14 model has therefore proven a valuable development tool for the SpiNNaker neuromorphic platform, providing a validated benchmark which could be used to analyze and unlock the potential of the SpiNNaker architecture. This research has been further developed by the SpiNNaker team to understand optimal application mapping to the neuromorphic system (eg Peres and Rhodes (2022)) and has helped inform the design of the next-generation neuromorphic system SpiNNaker 2 (Mayr et al. 2019), enabling the next step in computer architecture research.

### GPU-accelerated simulations

Thomas Nowotny elaborated on the impact of PD14 on the development of the GeNN (GPU-enhanced Neuronal Networks) software (Yavuz et al. 2016; Knight et al. 2021). After the first comparative benchmarks of PD14 on the SpiNNaker neuromorphic hardware and the NEST simulator on HPC were published by van Albada et al. (2018), it became clear that this model posed challenging problems for efficient simulations. The original SpiNNaker implementation took hours in initialization for minutes of actual simulation time. Diamond et al. (2016) had recognized this problem earlier, and the GeNN developers were completing improvements in GeNN that changed how models are initialized. Instead of determining detailed connectivity matrices in CPU space and copying them to the GPU, connectivity could now be specified with “connectivity code snippets,” which are run in parallel on the GPU to initialize the model. The large number of heterogeneous synapse populations of PD14 also motivated further optimization in GeNN that now automatically merges neuron and connection groups with the same underlying dynamical model to decrease compilation overheads and increase kernel performance.

Motivated by the results of the van Albada benchmark, Knight and Nowotny (2018) performed comparable benchmarks of PD14 in GeNN on several GPU platforms. They found that at the time, the GeNN simulation just beat the fastest runs in NEST on the Jülich HPC systems. The detailed documentation, availability of reference source code, and established validation methodology of PD14 greatly aided the ease of running a fair comparison in this work.

Since the first comparisons in 2018, subsequent benchmarks on improved software and hardware have seen an interesting neck-and-neck race between SpiNNaker, NEST on HPC, and GeNN on GPU (Rhodes et al. 2019; Kurth et al. 2022).

The later publication of the multi-area model that directly builds on PD14 (Schmidt et al. 2018a, 2018b) saw another round of innovation in GeNN simulation methodology. Because the synaptic connections were so numerous in the multi-area model that they could not be stored on a single GPU, James Knight rediscovered the idea of “procedural connectivity” (Roth et al. 1997; Izhikevich and Edelman 2008) where synaptic connections are not stored at all. Instead, the connectivity code snippets normally used for initialization are run whenever the existence and weight of a synapse need to be established. Combined with modern counter-based random number generators (Salmon et al. 2011), this allows very efficient simulations of extremely large networks in GeNN on individual GPUs (Knight and Nowotny 2021).

Nowotny concluded his contribution by presenting mlGeNN (https://github.com/genn-team/ml_genn), an interface that makes GeNN more accessible for machine learning applications (Turner et al. 2022). This new direction will directly benefit from the improvements that the PD14 simulator race has motivated.

### Optimizing NEST GPU

As a further development of simulation code for GPUs, Gianmarco Tiddia presented the GPU-based implementation of the NEST simulator and discussed how PD14 fostered its optimization. Indeed, the development of NEST GPU was motivated by the need to harness the computational power of GPUs while keeping the well-established NEST interface, enabling efficient and scalable large-scale neural simulations without compromising usability or compatibility with existing workflows. When the prototype library of NEST GPU was in development, SpiNNaker and GeNN developers had already employed PD14 for testing their simulators due to its complexity, which makes it an optimal benchmark for identifying the bottlenecks in the simulation of networks with natural connection density and realistic spiking activity. For this reason, PD14 was also implemented in NEST GPU (Golosio et al. 2021). The model is currently employed to test new versions of the code with an improved version of the protocol by van Albada et al. (2018) to verify the correctness of the simulation.

Tiddia then discussed the importance of initialization time for spiking network simulations, especially when performing a large number of simulations. The network construction algorithm of the prototype library, being performed on the CPU first, benefited from the standard C++ libraries. However, the initialization time was comparable to or larger than that for the CPU version of NEST, mainly because of the costly copying of connections from random access memory (RAM) to GPU memory. A novel algorithm proposed by Golosio et al. (2023) enabled the initialization of the simulator to be performed at runtime directly on the GPU, with an improvement on the order of 100 times with respect to the algorithm previously implemented. Also in this case, the model chosen for evaluating the performance of the novel initialization algorithm was PD14, which was also employed for validating the results of the simulator. NEST GPU is currently able to initialize at runtime the full-scale PD14 model in about 0.5 s on the data center GPU NVIDIA A100, with similar results achieved with the consumer GPU NVIDIA RTX 4090.

Tiddia concluded his presentation by showing the results achieved by NEST GPU in the simulation of the multi-area model by Schmidt et al. (2018b), which employs PD14 as a building block template (see section Building Block for Models with Larger  Explanatory Scope). In this case, because of the model size, a multi-GPU system is required to perform the simulation using NEST GPU. The GPU version of NEST outperforms the CPU version by a factor of three in terms of runtime (Tiddia et al. 2022), even though both use the message-passing interface MPI (Message Passing Interface Forum 2009) for the communication between compute nodes. One reason is the mapping of cortical areas to individual GPUs, thus decreasing the number of spikes that need to be delivered to neurons on other GPUs.

### The making of neuroAI^x^

Tobias Gemmeke presented the development of the neuroAI^x^ FPGA cluster, a platform for accelerating large-scale neuroscience simulations. The primary driver was a discussion with computational neuroscientists that expressed their need for simulations running faster than biological real-time to enable the analysis of long-term plasticity effects and broad parameter search. Additionally, core requirements for a dedicated neuroscience simulation platform in a research context were identified: flexibility, observability, scalability, and replicability (Kauth et al. 2023a). With these goals in mind, Kauth et al. (2020) explored in initial studies various existing communication schemes alongside novel concepts. To support the exploration with quantitative data, the neuroAI^x^ framework was created (Kauth et al. 2023a). It consists of three major pillars: (i) static simulation, ie statistical spike generation for fast system exploration, (ii) dynamic simulation, ie system behavior emulation for analyzing time-varying effects like increased network traffic on shared links, and (iii) a hardware emulator based on an FPGA cluster—actual neuronal computations for benchmarking, empirical fitting of the model, and refinement of the simulations. The emerging novel algorithms for communication, synchronization, and memory access, coupled with an efficient implementation of neuron models, resulted in the disruptive improvement of the neuroAI^x^ FPGA cluster (Kauth et al. 2023b).

neuroAI^x^ consists of 35 FPGAs, interconnected via high-speed small form-factor pluggable (SFP) and serialized AT attachment (SATA) links. The network topology and underlying routing algorithms were specifically developed for simulating biological neural networks with realistic connectomes. PD14 was a major driver behind this development for two reasons. Firstly, it exhibits realistic firing rate statistics at scale, thus imposing proper requirements on a simulator. Secondly, its clear definition, open-source implementation, and role in rich, state-of-the-art research make it a strong benchmark for comparing various solutions. It was demonstrated that neuroAI^x^ can simulate PD14 20 times faster than real-time—faster and more energy-efficient than any other solution so far (Kauth et al. 2023a). The results were verified against the PyNN implementation of PD14 and showed full correspondence, indicating the suitability of neuroAI^x^ as a viable neuroscience simulation platform.

After this successful proof-of-concept study, the development of a second-generation cluster neuroAI^x^ 2.0 was initiated. Based on more powerful modern FPGAs, this will not only allow larger networks at higher acceleration. Its release will also incorporate support for (three-factor) plasticity rules and a user-friendly cloud interface with NEST/NESTML integration. This will open up neuroAI^x^ to the neuroscience community, encouraging and supporting research on larger and more realistic models than ever before, for instance scaling PD14 up to multiple areas in the neocortex.

## Discussion

In the decade since the publication of the PD14 microcircuit model, neuroscience has addressed its software crisis (Aimone et al. 2023). Techniques for reliable software development have been developed and are now in widespread use. Recently, research software engineering (RSE) has emerged as a field (Felderer et al. 2025) and neuroscientists actively contribute to this endeavor. It has also become apparent that software has a much longer life cycle than hardware, which is typically replaced every 5 years. Some neuroscience simulation codes, including NEURON and NEST, have already been with us for some 30 years. This means that relevant scientific software needs to be operated and maintained as scientific infrastructure. Unfortunately, funding bodies have not yet fully realized this (Hocquet et al. 2024).

During the European Human Brain Project (HBP), participating scientists have developed a deeper appreciation of the formal separation between a particular neuroscientific model and a generic simulation engine (Einevoll et al. 2019): Simulation engines can be operated as an infrastructure and continuously optimized, while many network models can be explored with the same engine. As exemplified by PD14, neuronal network models can now remain relevant for more than a decade. At the same time, models are becoming so complex that they can no longer be expressed in a few dozen lines of code, see for example the multi-scale model of Schmidt et al. (2018a). Therefore, not only simulation engines but also brain models have today become scientific infrastructure.

Open repositories for models such as OSB, ModelDB, or the EBRAINS Knowledge Graph provide crucial infrastructure for sharing models and thus for the establishment of models as scientific infrastructure, while simulator-independent model specification languages such as PyNN, SONATA (Dai et al. 2020), or NeuroML support the separation of the model from the simulation engine. Just like simulation engines, these models need to be maintained in the long term, providing support for new users, ensuring efficient execution on evolving simulation software and hardware, and integration of new scientific insights. We believe that the computational neuroscience community will increasingly require and develop complex, shared models as research platforms. Solving the challenge of providing and funding maintenance for brain models and simulation machines will therefore be critical to sustained progress in computational neuroscience.

Experimental techniques have advanced tremendously since work on PD14 began and the resulting data are increasingly standardized (eg through Neurodata without Borders (https://nwb.org)) and shared via repositories such as the Distributed Archives for Neurophysiology Data Integration (DANDI, https://about.dandiarchive.org) and the EBRAINS Knowledge Graph (https://search.kg.ebrains.eu). Three-dimensional electron microscopy of brain tissue provides detailed information about the structure of the neuronal network (Arkhipov et al. 2025; Bae et al. 2025). This significantly reduces the need to integrate data obtained by widely differing methods to construct models such as PD14. Indeed, Kurth et al. (2025) recently tested hypotheses that Potjans and Diesmann (2014) had to make to enable data bridging against explicit data from the MICrONS project (Ding et al. 2025). Electrophysiology with hundreds of electrodes, optophysiology, as well as layer-resolved magnetic resonance imaging further constrain the interaction between cell types and areas. In light of these developments, we are optimistic that larger models with greater explanatory scope can be constructed.

Critique of PD14 has focused on two points: incorrect network dynamics and lack of function. In terms of detailed network dynamics, researchers noticed early on that PD14 exhibits much less power at low frequencies than observed in the brain and that PD14 may be less sensitive in its response to single spikes than actual brain networks. Also, some functional aspects seem to rely on the interplay of different inhibitory cell types, while the model collapses them to a single type. Models building on PD14 discussed here show that some of these limitations indeed disappear in larger structures. Research addressing observed differences between PD14 and actual brain dynamics can thus elucidate which aspects of a network are essential to explain its dynamics.

Concerning the perceived lack of function, PD14 was intentionally created as a generic model based on detailed anatomical data to complement the large number of network models engineered to implement specific functions. PD14 thus rather describes the ground state of dynamics and the response to perturbations than some concrete information processing step in the brain. The re-use of PD14 for a model of attention presented above shows that a general model such as PD14 can provide a useful starting point for modeling of brain function. An important next stage in brain modeling will be to expand models to cover the spatially organized representation of information in the brain and to close the major brain-scale functional circuits.

Recent advances have led to neuronal network models with internal structure validated on multiple levels against data sets such as MICrONS and an increasing ability to exhibit aspects of brain function. Such models bring us steadily closer to creating digital twins of brain components (Amunts et al. 2024). The term describes a virtual representation of a complex physical object such as a spacecraft or a production plant. The idea is that the digital twin captures physical constraints in sufficient detail that modifications can be planned and their consequences be observed before the physical object needs to be touched. Today, models based on artificial intelligence can capture structural and response properties of complex brain circuits from large data and can thus predict the outcome of new experiments (Wang et al. 2025). However, to find mechanistic explanations of brain function and thus deepen our scientific understanding of the brain, digital twins need to represent the anatomical and physiological constraints of the brain on multiple levels. Thus, the combination of artificial intelligence techniques and novel experimental techniques will enable the community to advance the fruitful interplay between top–down and down bottom–up modeling to a new level.

The largest obstacle to the widespread adoption of large brain models as research infrastructure may be educational and social. Many publications in computational neuroscience are still based on ad hoc models of a particular brain function without embedding in the larger tissue and not infrequently implemented from scratch in general-purpose programming languages (Senk et al. 2022), hampering model re-use. Indeed, some colleagues express an explicit preference for models they can simulate on their laptops. While small models have provided important insights, their explanatory scope is limited, and computational neuroscience stands to benefit from taking the step from individual lab bench to large-scale infrastructure. Several participants expressed the view that the international advanced courses in computational neuroscience have educated a new generation of researchers who use existing models as building blocks and skillfully use existing digital tools to address challenging questions. Installation-free solutions for the exploration of complex models are also far more powerful today than a decade ago (Senk et al. 2017). Today, among others, OSB and the EBRAINS Research Infrastructure provide powerful platforms for work with complex models in teaching and research. The situation is reversing: The desire to have full independence using the personal laptop only is for some young researchers even replaced by a feeling of awkwardness if software needs to be installed. Simulations small or large are regularly done in the cloud without any installation on the personal laptop or tablet using the same model description language. This removes technical and psychological barriers in the use of larger models.

We believe that computational neuroscience may be at a new dawn, the sometimes painful transition from a science where discoveries are made by individuals to a science where questions of greater complexity can be addressed by large-scale instruments, at the price of giving up some individuality (Galison 1997).

## References

[ref1] Aimone JB et al. 2023. Editorial: neuroscience, computing, performance, and benchmarks: why it matters to neuroscience how fast we can compute. Front Neuroinform. 17:1157418. 10.3389/fninf.2023.1157418. 37064716 PMC10098318

[ref2] Akaike H. 1973. Information theory and an extension of the maximum likelihood principle. In: Petrov BN and Caski F editors. 2nd International Symposium on Information Theory. Akademiai Kiado, Budapest. pp. 267–281.

[ref5] Albers J et al. 2022. A modular workflow for performance benchmarking of neuronal network simulations. Front Neuroinform. 16:837549. 10.3389/fninf.2022.837549. PMC9131021 35645755

[ref6] Amit DJ, Brunel N. 1997. Dynamics of a recurrent network of spiking neurons before and following learning. Netw Comput Neural Syst. 8:373–404. 10.1088/0954-898X_8_4_003.

[ref7] Amunts K et al. 2024. The coming decade of digital brain research: a vision for neuroscience at the intersection of technology and computing. Imaging Neurosci. 2:1–35. 10.1162/imag_a_00137. PMC12247565 40800542

[ref8] Antolik J et al. 2024. A comprehensive data-driven model of cat primary visual cortex. PLoS Comput Biol. 20:e1012342. 10.1371/journal.pcbi.1012342. 39167628 PMC11371232

[ref9] Arkhipov A et al. 2025. Integrating multimodal data to understand cortical circuit architecture and function. Nat Neurosci. 28:717–730. 10.1038/s41593-025-01904-7. 40128391

[ref10] Bae JA et al. 2025. Functional connectomics spanning multiple areas of mouse visual cortex. Nature. 640:435–447. 10.1038/s41586-025-08790-w. 40205214 PMC11981939

[ref11] Bastos AM et al. 2012. Canonical microcircuits for predictive coding. Neuron. 76:695–711. 10.1016/j.neuron.2012.10.038. 23177956 PMC3777738

[ref12] Bazhenov M, Timofeev I, Steriade M, Sejnowski TJ. 2002. Model of thalamocortical slow-wave sleep oscillations and transitions to activated states. J Neurosci. 22:8691–8704. 10.1523/JNEUROSCI.22-19-08691.2002. 12351744 PMC6757797

[ref13] Billeh YN et al. 2020. Systematic integration of structural and functional data into multi-scale models of mouse primary visual cortex. Neuron. 106:388–403.e18. 10.1016/j.neuron.2020.01.040. 32142648

[ref14] Bos H, Diesmann M, Helias M. 2016. Identifying anatomical origins of coexisting oscillations in the cortical microcircuit. PLoS Comput Biol. 12:e1005132. 10.1371/journal.pcbi.1005132. 27736873 PMC5063581

[ref15] Bower JM, Beeman D. 1998. The book of GENESIS: exploring realistic neural models with the GEneral NEural SImulation system. Springer-Verlag, New York, 10.1007/978-1-4612-1634-6.

[ref16] Brunel N . 2000. Dynamics of networks of randomly connected excitatory and inhibitory spiking neurons. J Physiol Paris. 94:445–463. 10.1016/s0928-4257(00)01084-6. 11165912

[ref17] Cain N, Iyer R, Koch C, Mihalas S. 2016. The computational properties of a simplified cortical column model. PLoS Comput Biol. 12:e1005045. 10.1371/journal.pcbi.1005045. 27617444 PMC5019422

[ref18] Cannon RC et al. 2014. LEMS: a language for expressing complex biological models in concise and hierarchical form and its use in underpinning NeuroML 2. Front Neuroinform. 8:79. 10.3389/fninf.2014.00079. 25309419 PMC4174883

[ref18a] Chu CCJ, Chien PF, Hung CP. 2014. Tuning dissimilarity explains short distance decline of spontaneous spike correlation in macaque V1. Vision Research. 96:113–132. 10.1016/j.visres.2014.01.008. 24486852

[ref20] Churchland MM et al. 2010. Stimulus onset quenches neural variability: a widespread cortical phenomenon. Nat Neurosci. 13:369–378. 10.1038/nn.2501. 20173745 PMC2828350

[ref21] Cordeiro VL . 2019. Models of neural networks with stochastic neurons and different topologies: construction and analysis [master's dissertation]. Universidade de Sao Paulo, Ribeirao Preto.

[ref22] Cuellar AA et al. 2003. An overview of CellML 1.1, a biological model description language. SIMULATION. 79:740–747. 10.1177/0037549703040939.

[ref23] Dai K et al. 2020. The SONATA data format for efficient description of large-scale network models. PLoS Comput Biol. 16:e1007696. 10.1371/journal.pcbi.1007696. 32092054 PMC7058350

[ref24] Dasbach S, Tetzlaff T, Diesmann M, Senk J. 2021. Dynamical characteristics of recurrent neuronal networks are robust against low synaptic weight resolution. Front Neurosci. 15:757790. 10.3389/fnins.2021.757790. PMC8740282 35002599

[ref25] Davison AP et al. 2009. PyNN: a common Interface for neuronal network simulators. Front Neuroinform. 2:11. 10.3389/neuro.11.011.2008. 19194529 PMC2634533

[ref55] de Kamps M et al. 2008. The state of MIIND. Neural Netw. 21:1164–1181. 10.1016/j.neunet.2008.07.006. 18783918

[ref26] De Schutter E . 2008. Why are computational neuroscience and systems biology so separate? PLoS Comput Biol. 4:e1000078. 10.1371/journal.pcbi.1000078. 18516226 PMC2367448

[ref27] Dentico D et al. 2014. Reversal of cortical information flow during visual imagery as compared to visual perception. NeuroImage. 100:237–243. 10.1016/j.neuroimage.2014.05.081. 24910071 PMC4310722

[ref28] Diamond A, Nowotny T, Schmuker M. 2016. Comparing neuromorphic solutions in action: implementing a bio-inspired solution to a benchmark classification task on three parallel-computing platforms. Front Neurosci. 9:491. 10.3389/fnins.2015.00491. PMC4705229 26778950

[ref29] Diesmann M . 2013. The road to brain-scale simulations on K. BioSupercomputing Newsletter. 8:8. http://www.csrp.riken.jp/BSNewsLetters/BSNvol8-1303/EN/report03.html.

[ref30] Diesmann M, Gewaltig M-O, Aertsen A. 1995. SYNOD: an environment for neural systems simulations - language Interf ace and tutorial. Weizmann Institute of Science. Rehovot, Israel.

[ref31] Ding Z et al. 2025. Functional connectomics reveals general wiring rule in mouse visual cortex. Nature. 640:459–469. 10.1038/s41586-025-08840-3. 40205211 PMC11981947

[ref32] Djurfeldt M et al. 2010. Run-time interoperability between neuronal network simulators based on the MUSIC framework. Neuroinformatics. 8:43–60. 10.1007/s12021-010-9064-z. 20195795 PMC2846392

[ref33] Douglas RJ, Martin KA. 2004. Neuronal circuits of the neocortex. Annu Rev Neurosci. 27:419–451. 10.1146/annurev.neuro.27.070203.144152. 15217339

[ref34] Einevoll GT et al. 2019. The scientific case for brain simulations. Neuron. 102:735–744. 10.1016/j.neuron.2019.03.027. 31121126

[ref35] Eppler J, Helias M, Muller E, Diesmann M, Gewaltig M-O. 2009. PyNEST: a convenient interface to the NEST simulator. Front Neuroinform. 2:12. 10.3389/neuro.11.012.2008. 19198667 PMC2636900

[ref36] Farley B, Clark W. 1954. Simulation of self-organizing systems by digital computer. Transactions of the IRE Professional Group on Information Theory. 4:76–84. 10.1109/TIT.1954.1057468.

[ref37] Felderer M et al. 2025. Investigating research software engineering: toward RSE research. Commun ACM. 68:20–23. 10.1145/3685265.

[ref38] Furber SB et al. 2013. Overview of the SpiNNaker system architecture. IEEE Trans Comput. 62:2454–2467. 10.1109/TC.2012.142.

[ref39] Galison P . 1997. Image and logic: a material culture of microphysics. The University of Chicago Press, Chicago and London.

[ref40] Gleeson P et al. 2010. NeuroML: a language for describing data driven models of neurons and networks with a high degree of biological detail. PLoS Comput Biol. 6:e1000815. 10.1371/journal.pcbi.1000815. 20585541 PMC2887454

[ref41] Gleeson P et al. 2019. Open source brain: a collaborative resource for visualizing, Analyzing, simulating, and developing standardized models of neurons and circuits. Neuron. 103:395–411.e5. 10.1016/j.neuron.2019.05.019. 31201122 PMC6693896

[ref42] Golosio B et al. 2021. Fast simulations of highly-connected spiking cortical models using GPUs. Front Comput Neurosci. 15:627620. 10.3389/fncom.2021.627620. PMC7925400 33679358

[ref43] Golosio B et al. 2023. Runtime construction of large-scale spiking neuronal network models on GPU devices. Appl Sci. 13:9598. 10.3390/app13179598.

[ref44] Habenschuss S, Jonke Z, Maass W. 2013. Stochastic computations in cortical microcircuit models. PLoS Comput Biol. 9:e1003311. 10.1371/journal.pcbi.1003311. 24244126 PMC3828141

[ref45] Haeusler S, Maass W. 2007. A statistical analysis of information-processing properties of lamina-specific cortical microcircuit models. Cereb Cortex. 17:149–162. 10.1093/cercor/bhj132. 16481565

[ref46] Heittmann A et al. 2022. Simulating the cortical microcircuit significantly faster than real time on the IBM INC-3000 neural supercomputer. Front Neurosci. 15:728460. 10.3389/fnins.2021.728460. PMC8811464 35126034

[ref47] Hill S, Tononi G. 2005. Modeling sleep and wakefulness in the thalamocortical system. J Neurophysiol. 93:1671–1698. 10.1152/jn.00915.2004. 15537811

[ref48] Hocquet A et al. 2024. Software in science is ubiquitous yet overlooked. Nat Comput Sci. 4:465–468. 10.1038/s43588-024-00651-2. 38951645

[ref49] Howell F et al. 2003. Linking computational neuroscience simulation tools—a pragmatic approach to component-based development. Neurocomputing. 52-54:289–294. 10.1016/S0925-2312(02)00781-6.

[ref50] Hucka M et al. 2003. The systems biology markup language (SBML): a medium for representation and exchange of biochemical network models. Bioinformatics. 19:524–531. 10.1093/bioinformatics/btg015. 12611808

[ref51] Hüllermeier E, Waegeman W. 2021. Aleatoric and epistemic uncertainty in machine learning: an introduction to concepts and methods. Mach Learn. 110:457–506. 10.1007/s10994-021-05946-3.

[ref52] Iyer R, Menon V, Buice M, Koch C, Mihalas S. 2013. The influence of synaptic weight distribution on neuronal population dynamics. PLoS Comput Biol. 9:e1003248. 10.1371/journal.pcbi.1003248. 24204219 PMC3808453

[ref53] Izhikevich EM, Edelman GM. 2008. Large-scale model of mammalian thalamocortical systems. Proc Natl Acad Sci USA. 105:3593–3598. 10.1073/pnas.0712231105. 18292226 PMC2265160

[ref54] Kamiji NL et al. 2017 Nov 11–15. A cortical microcircuit model with heterogeneous excitatory and inhibitory neurons. Poster presented at: Neuroscience 2017. Washington, DC.

[ref56] Kauth K, Stadtmann T, Brandhofer R, Sobhani V, Gemmeke T. 2020. Communication architecture enabling 100x accelerated simulation of biological neural networks. In: Proceedings of the Workshop on System-Level Interconnect: Problems and Pathfinding Workshop. San Diego, California: Association for Computing Machinery. Article 2.

[ref57] Kauth K, Stadtmann T, Sobhani V, Gemmeke T. 2023a. neuroAIx-framework: design of future neuroscience simulation systems exhibiting execution of the cortical microcircuit model 20x faster than biological real-time. Front Comput Neurosci. 17:1144143. 10.3389/fncom.2023.1144143. PMC10156974 37152299

[ref58] Kauth K, Stadtmann T, Sobhani V, Gemmeke T. 2023b. neuroAIx: FPGA cluster for reproducible and accelerated neuroscience simulations of SNNs, 2023 IEEE Nordic circuits and systems conference (NorCAS) Aalborg, Denmark, pp. 1–7. 10.1109/NorCAS58970.2023.10305473.

[ref59] Kiureghian AD, Ditlevsen O. 2009. Aleatory or epistemic? Does it matter? Struct Saf. 31:105–112. 10.1016/j.strusafe.2008.06.020.

[ref60] Knight JC, Nowotny T. 2018. GPUs outperform current HPC and neuromorphic solutions in terms of speed and energy when simulating a highly-connected cortical model. Front Neurosci. 12:941. 10.3389/fnins.2018.00941. PMC6299048 30618570

[ref61] Knight JC, Nowotny T. 2021. Larger GPU-accelerated brain simulations with procedural connectivity. Nat Comput Sci. 1:136–142. 10.1038/s43588-020-00022-7. 38217218

[ref62] Knight JC, Komissarov A, Nowotny T. 2021. PyGeNN: a python library for GPU-enhanced neural networks. Front Neuroinform. 15:659005. 10.3389/fninf.2021.659005. PMC8100330 33967731

[ref63] Kurth AC, Senk J, Terhorst D, Finnerty J, Diesmann M. 2022. Sub-realtime simulation of a neuronal network of natural density. Neuromorphic Comput Eng. 2:021001. 10.1088/2634-4386/ac55fc.

[ref64] Kurth A, Albers J, Diesmann M, van Albada S. 2025. Cell-type specific projection patterns promote balanced activity in cortical microcircuit s [preprint]. bioRxiv. 10.1101/2024.10.03.616539.

[ref65] Lansner A, Diesmann M. 2012. Virtues, pitfalls, and methodology of neuronal network Modeling and simulations on supercomputers. In: Le Novère N editor. Computational systems neurobiology Springer Netherlands, Dordrecht, pp. 283–315 10.1007/978-94-007-3858-4_10.

[ref66] Layer M et al. 2022. NNMT: mean-field based analysis tools for neuronal network models. Front Neuroinform. 16:835657. 10.3389/fninf.2022.835657. 35712677 PMC9196133

[ref67] Ling S, Liu T, Carrasco M. 2009. How spatial and feature-based attention affect the gain and tuning of population responses. Vis Res. 49:1194–1204. 10.1016/j.visres.2008.05.025. 18590754 PMC2696585

[ref68] Markram H et al. 2015. Reconstruction and simulation of neocortical microcircuitry. Cell. 163:456–492. 10.1016/j.cell.2015.09.029. 26451489

[ref69] Martinez-Trujillo JC, Treue S. 2004. Feature-based attention increases the selectivity of population responses in primate visual cortex. Curr Biol. 14:744–751. 10.1016/j.cub.2004.04.028. 15120065

[ref70] Mayr C, Hoeppner S, Furber S. 2019. SpiNNaker 2: a 10 million Core processor system for brain simulation and machine learning. In: Broenink JF et al editors. Communicating Process Architectures 2017 & 2018. IOS Press. pp 277–280. 10.3233/978-1-61499-949-2-277.

[ref71] McAdams CJ, Maunsell JHR. 1999. Effects of attention on orientation-tuning functions of single neurons in macaque cortical area V4. J Neurosci. 19:431–441. 10.1523/JNEUROSCI.19-01-00431.1999. 9870971 PMC6782389

[ref72] McDougal RA et al. 2017. Twenty years of ModelDB and beyond: building essential modeling tools for the future of neuroscience. J Comput Neurosci. 42:1–10. 10.1007/s10827-016-0623-7. 27629590 PMC5279891

[ref73] Message Passing Interface Forum . 2009. MPI: a message-passing Interface standard. 2.2, https://www.mpi-forum.org/docs/mpi-2.2/mpi22-report.pdf.

[ref74] Meier K . 2015. Brain-inspired multiscale computation in neuromorphic hybrid systems (BrainScaleS) project (European Union 7th Framework Program under grant agreement no. 269921), 4th periodic report (2014–2015). https://brainscales.kip.uni-heidelberg.de/public/results/BrainScaleS_269921_4thReport_PublishableSummary.pdf. Kirchhoff Institute for Physics, Heidelberg.

[ref75] Muller E et al. 2015. Python in neuroscience. Front Neuroinform. 9:11. 10.3389/fninf.2015.00011. 25926788 PMC4396193

[ref76] Omar C, Aldrich J, Gerkin RC. 2014. Collaborative infrastructure for test-driven scientific model validation. In: 36th International Conference on Software Engineering (ICSE), Hyderabad, India. Association for Computing Machinery, New York, NY. p. 524–527. 10.1145/2591062.2591129.

[ref77] Osborne H, Deutz L, de Kamps M. 2022. Multidimensional dynamical systems with noise. In: Computational modelling of the brain: modelling approaches to cells, circuits and networks Giugliano M, Negrello M, Linaro D editors. Springer International Publishing, Cham, p. 159–178. 10.1007/978-3-030-89439-9_7.

[ref78] Peres L, Rhodes O. 2022. Parallelization of neural processing on neuromorphic hardware. Front Neurosci. 16:867027. 10.3389/fnins.2022.867027. 35620669 PMC9128596

[ref79] Potjans TC, Diesmann M. 2008 15–19 Nov. Consistency of in vitro and in vivo connectivity estimates: Statistical assessment and application to cortical network modeling. Poster presented at: Neuroscience 2008. Washington, DC.

[ref80] Potjans TC, Diesmann M. 2014. The cell-type specific cortical microcircuit: relating structure and activity in a full-scale spiking network model. Cereb Cortex. 24:785–806. 10.1093/cercor/bhs358. 23203991 PMC3920768

[ref81] Pronold J, Morales-Gregorio A, Rostami V, van Albada S. 2024a. Cortical multi-area model with joint excitatory-inhibitory clusters accounts for spiking statistics, inter-area propagation, and variability dynamic s [preprint]. bioRxiv. 10.1101/2024.01.30.577979.

[ref82] Pronold J et al. 2024b. Multi-scale spiking network model of human cerebral cortex. Cereb Cortex. 34:bhae409. 10.1093/cercor/bhae409. PMC11491286 39428578

[ref83] René A, Longtin A. 2025. Selecting fitted models under epistemic uncertainty using a stochastic process on quantile functions. Nat Commun. 16:9393. 10.1038/s41467-025-64658-7. PMC12549851 41130986

[ref84] René A, Longtin A, Macke JH. 2020. Inference of a mesoscopic population model from population spike trains. Neural Comput. 32:1448–1498. 10.1162/neco_a_01292. 32521212

[ref85] Reynolds JH, Chelazzi L, Desimone R. 1999. Competitive mechanisms subserve attention in macaque areas V2 and V4. J Neurosci. 19:1736–1753. 10.1523/JNEUROSCI.19-05-01736.1999. 10024360 PMC6782185

[ref86] Rhodes O et al. 2019. Real-time cortical simulation on neuromorphic hardware. Philos Trans R Soc A Math Phys Eng Sci. 378:20190160. 10.1098/rsta.2019.0160. PMC6939236 31865885

[ref87] Romaro C, Najman FA, Lytton WW, Roque AC, Dura-Bernal S. 2021. NetPyNE implementation and scaling of the Potjans-Diesmann cortical microcircuit model. Neural Comput. 33:1993–2032. 10.1162/neco_a_01400. 34411272 PMC8382011

[ref88] Roque AC et al. 2017 Nov 11–15. A stochastic cortical microcircuit model. Poster presented at: Neuroscience 2017, Washington, DC.

[ref89] Rostami V et al. 2024. Spiking attractor model of motor cortex explains modulation of neural and behavioral variability by prior target information. Nat Commun. 15:6304. 10.1038/s41467-024-49889-4. 39060243 PMC11282312

[ref90] Roth U, Eckardt F, Jahnke A, Klar H. 1997. Efficient on-line computation of connectivity: architecture of the connection unit of NESPINN. In: Klar H, editor. Proceedings of the 6th International Conference on Microelectronics for Neural Networks, Evolutionary & Fuzzy Systems (MicroNeuro '97). Technische Universität Dresden, Dresden, Germany. pp 31–39.

[ref91] Salmon JK, Moraes MA, Dror RO, Shaw DE. 2011. Parallel random numbers: as easy as 1, 2, 3. In: Proceedings SC 11 International Conference for High Performance Computing, Networking, Storage and Analysis. Seattle, Washington: Association for Computing Machinery, NY. Article no 16, p 1–12. 10.1145/2063384.2063405.

[ref92] Schmidt M, Bakker R, Hilgetag CC, Diesmann M, van Albada SJ. 2018a. Multi-scale account of the network structure of macaque visual cortex. Brain Struct Funct. 223:1409–1435. 10.1007/s00429-017-1554-4. 29143946 PMC5869897

[ref93] Schmidt M et al. 2018b. A multi-scale layer-resolved spiking network model of resting-state dynamics in macaque visual cortical areas. PLoS Comput Biol. 14:e1006359. 10.1371/journal.pcbi.1006359. 30335761 PMC6193609

[ref94] Schuecker J, Schmidt M, van Albada SJ, Diesmann M, Helias M. 2017. Fundamental activity constraints lead to specific interpretations of the connectome. PLoS Comput Biol. 13:e1005179. 10.1371/journal.pcbi.1005179. 28146554 PMC5287462

[ref95] Schwalger T . 2021. Mapping input noise to escape noise in integrate-and-fire neurons: a level-crossing approach. Biol Cybern. 115:539–562. 10.1007/s00422-021-00899-1. 34668051 PMC8551127

[ref96] Schwalger T, Deger M, Gerstner W. 2017. Towards a theory of cortical columns: from spiking neurons to interacting neural populations of finite size. PLoS Comput Biol. 13:e1005507. 10.1371/journal.pcbi.1005507. 28422957 PMC5415267

[ref97] Schwarz G . 1978. Estimating the dimension of a model. Ann Stat. 6:461–464.

[ref98] Senk J et al. 2017. A collaborative simulation-analysis workflow for computational neuroscience using HPC, high-performance scientific computing. Springer International Publishing, Cham, pp 243–256.

[ref99] Senk J et al. 2022. Connectivity concepts in neuronal network modeling. PLoS Comput Biol. 18:e1010086. 10.1371/journal.pcbi.1010086. 36074778 PMC9455883

[ref100] Senk J et al. 2025. Constructive community race: full-density spiking neural network model drives neuromorphic computing [preprint]. arXiv. 10.48550/arXiv.2505.21185.

[ref101] Shimoura RO . 2021. Computational study of thalamocortical interactions: simulating oscillatory activity [doctoral dissertation]. USP Open Access Repository: University of São Paulo, Ribeirão Preto.

[ref102] Shimoura RO et al. 2018. [Re] the cell-type specific cortical microcircuit: relating structure and activity in a full-scale spiking network model. ReScience. 4:#2. 10.5281/zenodo.1244116. PMC3920768 23203991

[ref103] Teeter C et al. 2018. Generalized leaky integrate-and-fire models classify multiple neuron types. Nat Commun. 9:709. 10.1038/s41467-017-02717-4. 29459723 PMC5818568

[ref104] Tiddia G et al. 2022. Fast simulation of a multi-area spiking network model of macaque cortex on an MPI-GPU cluster. Front Neuroinform. 16:883333. 10.3389/fninf.2022.883333. PMC9289599 35859800

[ref105] Traub RD et al. 2005. Single-column thalamocortical network model exhibiting gamma oscillations, sleep spindles, and epileptogenic bursts. J Neurophysiol. 93:2194–2232. 10.1152/jn.00983.2004. 15525801

[ref106] Turner JP, Knight JC, Subramanian A, Nowotny T. 2022. mlGeNN: accelerating SNN inference using GPU-enabled neural networks. Neuromorphic Comput Eng. 2:024002. 10.1088/2634-4386/ac5ac5.

[ref3] van Albada S, Helias M, Diesmann M. 2015. Scalability of asynchronous networks is limited by one-to-one mapping between effective connectivity and correlations. PLoS Comput Biol. 11:e1004490. 10.1371/journal.pcbi.1004490. 26325661 PMC4556689

[ref4] van Albada SJ et al. 2018. Performance comparison of the digital neuromorphic hardware SpiNNaker and the neural network simulation software NEST for a full-scale cortical microcircuit model. Front Neurosci. 12:291. 10.3389/fnins.2018.00291. 29875620 PMC5974216

[ref108] van Vreeswijk C, Sompolinsky H. 1996. Chaos in neuronal networks with balanced excitatory and inhibitory activity. Science. 274:1724–1726. 10.1126/science.274.5293.1724. 8939866

[ref107] Vehtari A, Gelman A, Gabry J. 2017. Practical Bayesian model evaluation using leave-one-out cross-validation and WAIC. Stat Comput. 27:1413–1432. 10.1007/s11222-016-9696-4.

[ref109] Wagatsuma N, Potjans TC, Diesmann M, Fukai T. 2011. Layer-dependent attentional processing by top-down signals in a visual cortical microcircuit model. Front Comput Neurosci. 5:31. 10.3389/fncom.2011.00031. 21779240 PMC3134838

[ref110] Wagatsuma N, Potjans TC, Diesmann M, Sakai K, Fukai T. 2013. Spatial and feature-based attention in a layered cortical microcircuit model. PLoS One. 8:e80788. 10.1371/journal.pone.0080788. 24324628 PMC3855641

[ref111] Wagatsuma N, Nobukawa S, Fukai T. 2023. A microcircuit model involving parvalbumin, somatostatin, and vasoactive intestinal polypeptide inhibitory interneurons for the modulation of neuronal oscillation during visual processing. Cereb Cortex. 33:4459–4477. 10.1093/cercor/bhac355. 36130096 PMC10110453

[ref112] Waltemath D et al. 2011. Reproducible computational biology experiments with SED-ML--the simulation experiment description markup language. BMC Syst Biol. 5:198. 10.1186/1752-0509-5-198. 22172142 PMC3292844

[ref113] Wang EY et al. 2025. Foundation model of neural activity predicts response to new stimulus types. Nature. 640:470–477. 10.1038/s41586-025-08829-y. 40205215 PMC11981942

[ref114] Wilkinson MD et al. 2016. The FAIR guiding principles for scientific data management and stewardship. Sci Data. 3:160018. 10.1038/sdata.2016.18. 26978244 PMC4792175

[ref115] Womelsdorf T, Fries P, Mitra PP, Desimone R. 2006. Gamma-band synchronization in visual cortex predicts speed of change detection. Nature. 439:733–736. 10.1038/nature04258. 16372022

[ref116] Yavuz E, Turner J, Nowotny T. 2016. GeNN: a code generation framework for accelerated brain simulations. Sci Rep. 6:18854. 10.1038/srep18854. 26740369 PMC4703976

